# Spatial variability of heavy metals concentrations in soil of auto-mechanic workshop clusters in Nsukka, Nigeria

**DOI:** 10.1038/s41598-024-60044-3

**Published:** 2024-04-27

**Authors:** Stellamaris Chinenye Duru, Emmanuel Amagu Echiegu, Chinenye C. Anyadike, George Uwadiegwu Alaneme, Michael Emeka Okechukwu

**Affiliations:** 1 Agricultural and Bioresources Engineering Department, University of Nigeria, Nsukka, Nigeria; 2 Civil Engineering Department, Kampala International University, Kampala, Uganda

**Keywords:** Heavy metal concentration, Auto mechanics workshop cluster, Spent engine oil, Spatial maps, Environmental sciences, Engineering

## Abstract

The indiscriminate disposal of spent engine oils and other hazardous waste at auto mechanic workshops clusters in Nsukka, Enugu State, Nigeria is an environmental concern. This study examines the concentration of heavy metals in the soil inside the workshop cluster and in the unpolluted soil outside the workshop cluster at approximately 100 m. Ten sampling points were randomly selected from within the cluster and another ten from outside the cluster. Using a hand-held Global Positioning System, the coordinates of the selected points were established and used to create a digital map. Soil samples at depths of 0–30 cm and 30–60 cm, were analyzed for Cu, Fe, Zn, Pb, As and Cd using Spectrophotometer. Moisture content determination and particle size analysis were also done on the samples. Spatial variability of heavy metals concentrations of the studied site was also mapped with ArcGIS 10.2.2 using interpolation methods. Results showed that the soil ranged from sandy loam to sandy clay loam. Cadmium and Zinc had the lowest and highest concentration, respectively, in the studied area. Comparing the concentrations of heavy metals in soils within and outside the auto mechanic cluster revealed notable differences across various depths (0–30 cm and 30–60 cm). The analysis results for soil samples within the cluster exhibited concentration levels (mg/kg) ranging from 0.716–0.751 (Cu), 2.981–3.327 (Fe), 23.464–30.113 (Zn), 1.115–1.21 (Pb), 2.6–2.912 (As), and 0.133–0.365 (Cd) demonstrating a variation pattern in the order of Zn > Fe > As > Pb > Cu > Cd. Conversely, for soil samples outside the cluster, concentration levels (mg/kg) ranged from 0.611–0.618 (Cu), 2.233–2.516 (Fe), 12.841–15.736 (Zn), 0.887–0.903 (Pb), 1.669–1.911 (As), and 0.091–0.091 (Cd). To assess the disparity in heavy metal concentration levels between samples collected within and outside the clusters, ANOVA test was performed. The test showed significant difference in heavy metal concentrations between samples within and outside the auto mechanic cluster (*p* < 0.05), implying auto mechanic activities significantly impact heavy metal levels within the cluster compared to outside areas. The assessment of soil pollution utilized indices including the Geo-accumulation Index (Igeo), Contamination factor (Cf), and anthropogenic metal concentration (QoC). Zinc, Cadmium, and Arsenic showed the highest contamination factors, indicating significant soil contamination likely due to anthropogenic activities. The concentrations of the metals analyzed were within WHO permissible limits while the metals concentrations were also observed to decrease as depth was increased. Using ArcGIS 10.2.2, spatial maps showing heavy metal distribution were developed, with the Kriging method proving superior. This study suggests that heavy metal levels in the soil at the area be monitored on a regular basis.

## Introduction

The evaluation of heavy metals in soils around auto-mechanic workshop clusters in Nsukka, Nigeria, is essential due to potential environmental and human health risks associated with exposure to these contaminants^1^. It is generally accepted that soil is important for the survival of life in Earth's ecosystems, and its productivity as a medium for plants growth is very important to the survival of mankind^2^. Soil may be contaminated by the accumulation of heavy metal via the disposal of high-metal wastes, coal residues, and fertilizer application on land, pesticides, and other anthropogenic activities^3^. The activities of auto mechanic clusters, according to Adewole and Uchegbu^4^, are one of the most important sources of increased heavy metal concentration in the ecosystem.

Automobile repair/workshop activities are on the rise in Nigeria and most developing countries. The indiscriminate dumping of waste from these vehicles' maintenance and repairs has exacerbated the issue of soil pollution in most cities^5^. Auto-mechanic workshops often handle various hazardous materials, including fuels, lubricants, used batteries, asbestos from brake pads, oxidation materials and metals, which can contaminate surrounding soils through spills, leaks, and improper waste disposal practices^6,7^. As a result, heavy metals such as lead, cadmium, chromium, and mercury may accumulate in the soil, posing risks to ecosystems and human populations residing in the vicinity. Lack of adequate regulations and monitoring of the activities contributes to increased levels of heavy metals and hydrocarbons in the environment^8^.

Lenntech^9^ and Skaldina and Sorvari^10^, defined heavy metals as metallic chemical element with a relatively high density, which are toxic or poisonous at low concentrations. Its toxicity, as well as the danger it poses to human life and the environment, is a major source of concern^11^. Heavy metals and other contaminants, such as polycyclic aromatic hydrocarbons, are major components of petroleum hydrocarbons, which are used to manufacture the majority of materials used in maintaining and repairing vehicles^12^. Cadmium (Cd), copper (Cu), chromium (Cr), lead (Pb), manganese (Mn), nickel (Ni), and zinc (Zn) are non-biodegradable in the soil and are commonly used as additives in lubricants and gasoline.

Toxic heavy metals and hydrocarbons (HCs) coexist in many of the sites polluted by auto-mechanics activities across Nigeria and other developing countries, posing a serious threat to human health^13^. Indeed, the importance of trace elements in soil chemistry is becoming a global concern, particularly because soil is such an important component of both rural and urban environments. High levels of heavy metals in soils may not be due to anthropogenic effects, but rather to diagenetic causes^14,15^. Since metals from both natural and anthropogenic sources accumulate in soils, determining what fraction of the metal load comes from which source can be difficult. Sharma and Reddy^16^, mentioned soil washing and bioremediation as one of the few techniques of treating mixed wastes. As stated by Akpovata et al.^17^, the capacity of soil to retain introduced substances such as heavy metals rely on its sorption characteristics, including soil texture, pH, moisture content, and cation exchange capacity. Metals present on the soil surface can be transported to groundwater through runoff water^18^.

The assessment of heavy metals in soils serves multiple purposes, including environmental monitoring, risk assessment, and regulatory compliance. Understanding the extent and distribution of heavy metal contamination can help identify potential sources, assess environmental impacts, and develop mitigation strategies to protect soil quality and human health^19,20^. Additionally, such studies can inform policymakers, regulatory agencies, and community stakeholders about the need for remediation measures and regulatory enforcement to mitigate soil contamination and associated risks. The research carried out by Joseph et al.^21^ aimed to assess heavy metal contamination in soils around auto-mechanic workshops in Okitipupa, Ondo State, Nigeria. The study identified heavy metal presence, analyzed spatial distribution, and evaluated associated environmental and health risks. Results showed elevated heavy metal concentrations, particularly near workshop clusters, highlighting potential environmental and health hazards. The study emphasized the need for pollution control and remediation measures to mitigate risks and protect environmental and human health. Also, Osakwe^22^, assessed heavy metal contamination and characterize soil properties in automobile workshop areas in Abraka, Delta State, Nigeria. The research found elevated heavy metal concentrations, including lead, cadmium, chromium, and zinc, in soil samples. Variations in soil properties such as pH and organic matter content were observed. The study highlighted potential environmental and health risks associated with heavy metal contamination and emphasized the need for pollution control and remediation measures to mitigate these risks.

The identified gaps in the literature on the spatial variability of heavy metals concentrations in soil obtained from auto-mechanic workshop clusters in Nsukka, Nigeria, include inadequate application of advanced spatial analysis techniques, lack of depth-specific analysis, limited comparative studies between workshop clusters and surrounding areas, gaps in the application of pollution indices, and insufficient research on remediation strategies. Addressing these gaps through comprehensive research can provide valuable insights for developing effective pollution control measures and remediation strategies tailored to the specific characteristics of the study area^3,23^.

This study main idea in this research study is to investigate the presence and levels of heavy metals in soils around auto-mechanic workshop clusters in Nsukka, Nigeria, using a combination of field sampling, laboratory analysis, and spatial mapping techniques. The research goals include developing a digital map of the study area, randomly selecting soil sampling points using GPS, determining heavy metal concentrations at different depths within and outside workshop clusters, comparing pollution levels with standards, estimating pollution extent using indices, and creating spatial variability maps using ArcGIS. Through these objectives, the study aims to understand heavy metal contamination patterns and inform remediation efforts. By evaluating the spatial distribution and concentrations of heavy metals in soils, this research seeks to identify potential hotspots of contamination, assess the extent of environmental impacts, and provide insights into the factors influencing soil pollution in the study area. Furthermore, the findings of this study can contribute to informed decision-making processes for pollution control and remediation strategies, ultimately contributing to the sustainable management of soil resources and the protection of public health in Nsukka, Nigeria and similar urban settings with auto-mechanic workshop clusters. The study on the spatial variability of heavy metal concentrations in soil within auto-mechanic workshop clusters in Nsukka, Nigeria, holds significant importance. It provides valuable insights into environmental health risks, raises community awareness, informs policy development, assesses economic implications, and advances scientific knowledge. By quantifying heavy metal pollution and understanding its spatial distribution, the study contributes to efforts aimed at promoting environmental sustainability and protecting human health^24,25^.

## Materials and methods

### Study area description

**Figure 1:**
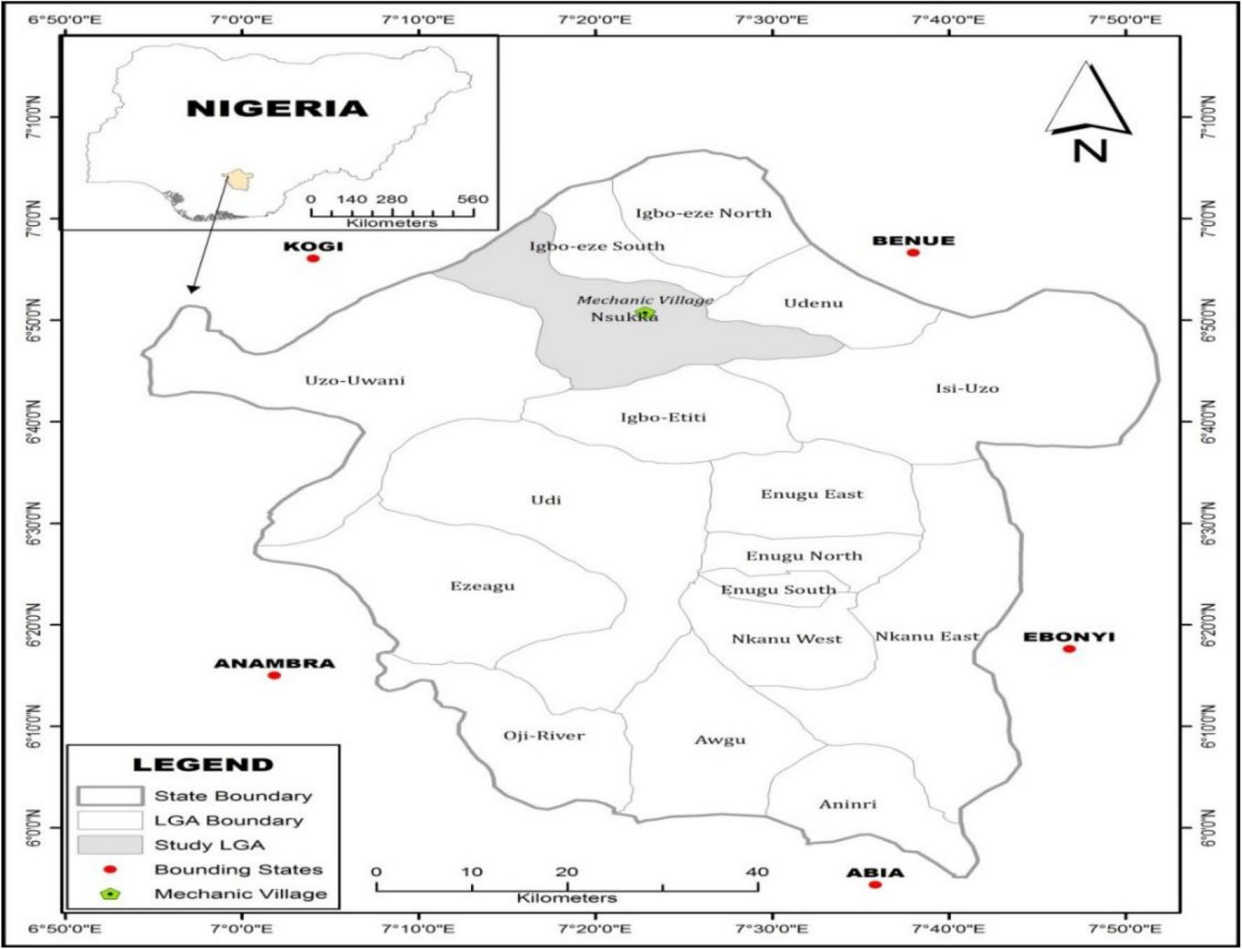
Map of Enugu State showing Nsukka.

**Figure 2:**
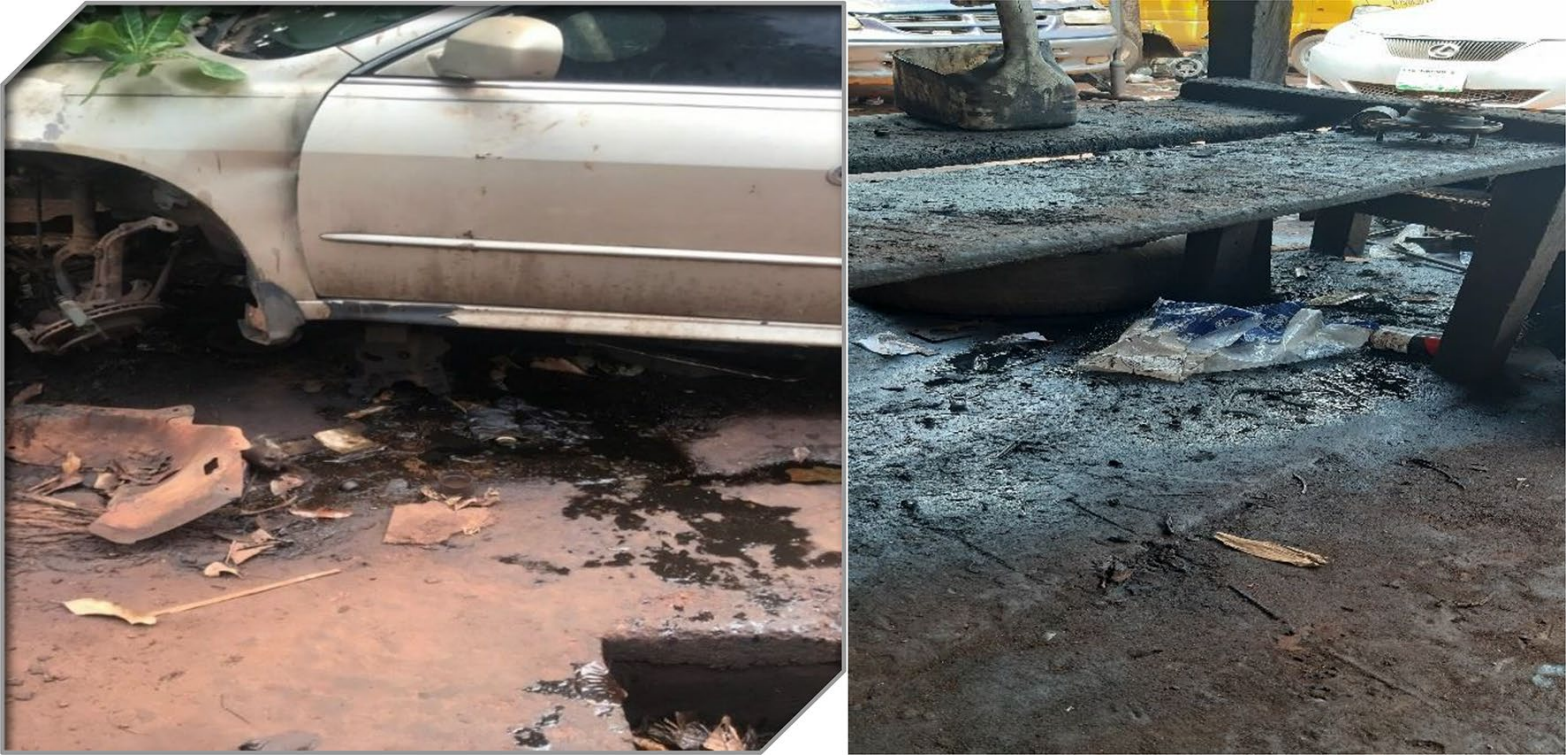
Oil spill at the study site.

Nsukka, a town and Local Government Area in Enugu State, Southeast Nigeria, covers an area of 1810 km^2^ and had a population of 309,633 in the 2006 census. Positioned at approximately 6.87°N latitude and 7.38°E longitude, Nsukka experiences a Tropical wet and dry or savanna climate and it lies at an elevation of None meters above sea level as shown in Fig. 1. The district's annual temperature averages 29.56 °C (85.21 °F), slightly higher than Nigeria's averages, with about 165.79 mm (6.53 inches) of precipitation and 201.51 rainy days annually. Nsukka boasts a humidity level of 74.13% and receives 10.5 sunshine hours. The town thrives with bustling economic activities, notably the Mechanic Village located along Ogurugu road, which has been servicing cars for over 30 years, catering to both local and non-local customers. Some pictures of the activities of the auto mechanic workshop are presented on Fig. 2.

### Soil sampling and preservation

**Figure 3:**
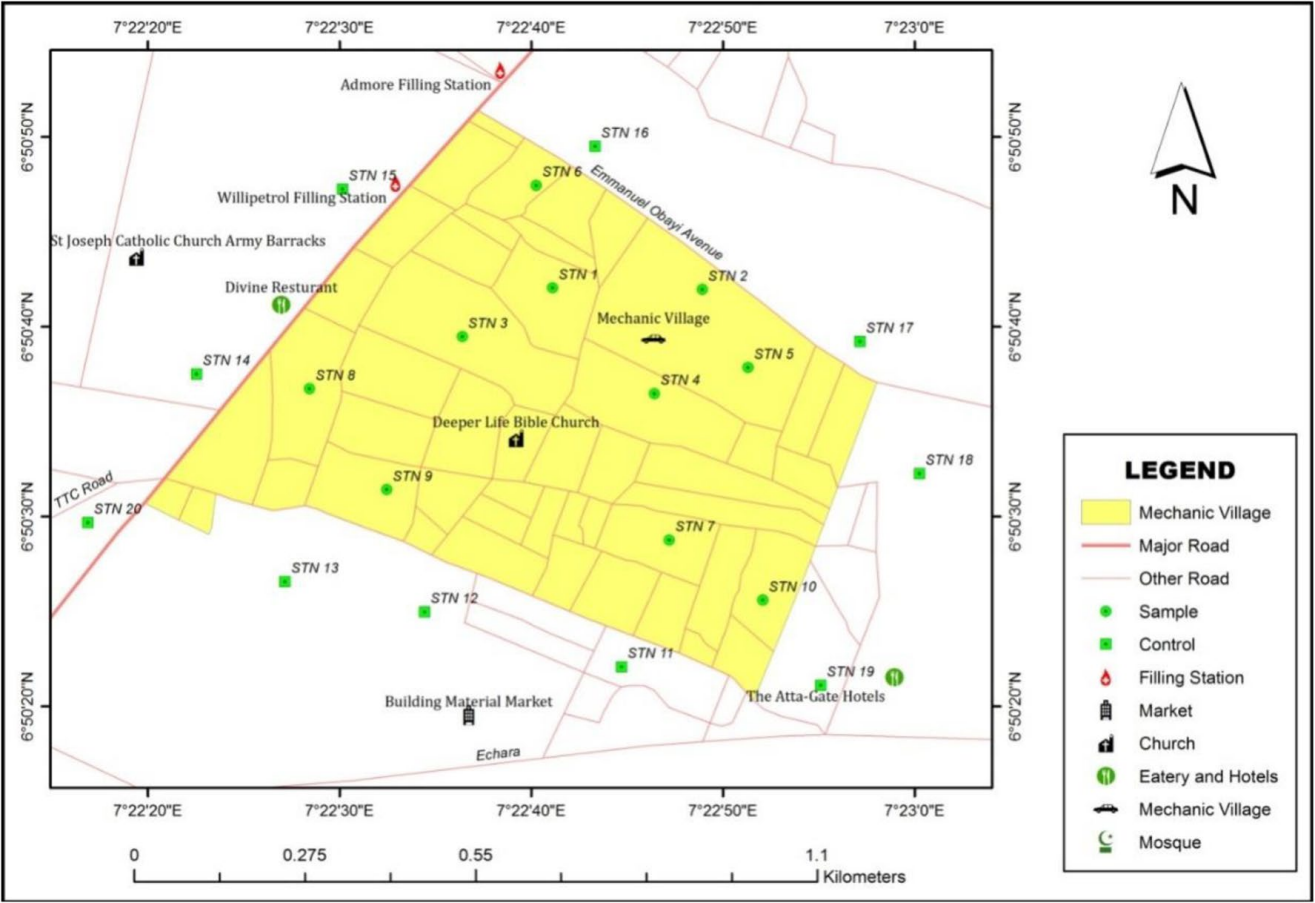
The sampling points in the study area generated with the aid of ArcGIS Software.

Soil sampling and preservation are critical processes in environmental monitoring, ensuring accurate analysis and informed decision-making. This involves planning sampling, collecting, preserving, and transporting samples securely to labs^26,27^. The soil sampling points were evenly and randomly selected to cover in and around the mechanic workshops cluster in Nsukka (Fig. 3). Ten (10) soil sampling points were selected within the cluster, while another ten (10) sampling points were selected from a control site (where neither vehicle repairs, industrial nor commercial activities are carried out) which is about 100 m from the workshop cluster. The first set of 10 samples were collected from a depth of 0 to 30 cm within the cluster while the second set were collected at the depth of 30 to 60 cm at the same sampling location where the first sets were collected. Other sets of ten samples each were also taken at 0–30 cm and 30–60 cm depth, respectively from points outside the cluster (the Control). A total of forty (40) soil samples were collected from the twenty (20) sampling points within and outside the mechanic village using soil auger.

The various coordinate values were collected from the sampled points within the study area using the GPS Device (Germin Etrex 30). The collected coordinate points of Latitude and Longitude using the ArcGIS software 10.2.2 were used to determine the various geospatial locations of the sample points over the study area and a geospatial map of the study area was produced. As stated earlier, a maximum of 10 sample points each were collected within and outside the mechanic village environment. This was to enable us to compare the variation in the solid mineral contents and propose a possible suitability class for the environment and the possible harm posed by the auto-mechanics’ activities and automobile waste being generated within the study area^28^. Soil samples were collected from the two depths using a soil auger. After each sampling, the soil auger was cleaned and washed with clean distilled water. Each sample was transferred to a labelled p**o**lythene container to avoid loss of moisture. The sampled soils were properly labeled in polythene and transported to the laboratory for soil moisture content and particle size analysis^29,30^.

### Determination of soil moisture content

The moisture content of the soil samples was determined using the gravimetric method. The initial weights (*w*_*1*_) of the soil samples were determined using a digital balance (OHAUS Model CP214). The samples were heated in an oven for 24 h at the temperature of 105°C and was observed to have dried after attaining a constant weight. Having known initial weight (*w*_*1*_) of the samples after collection from the field, the final weight (*w*_*2*_) after drying was subtracted from the initial weight (*w*_*1*_). Equation 1 was used to calculate the moisture content (MC)^31^.

1 $$MC = \frac{w_1 - w_2 }{{w_1 }} \times 100$$

where *MC* = moisture content; $w_1 ,w_2$ are the initial and final moisture content, respectively.

### Soil particle size distribution

**Figure 4:**
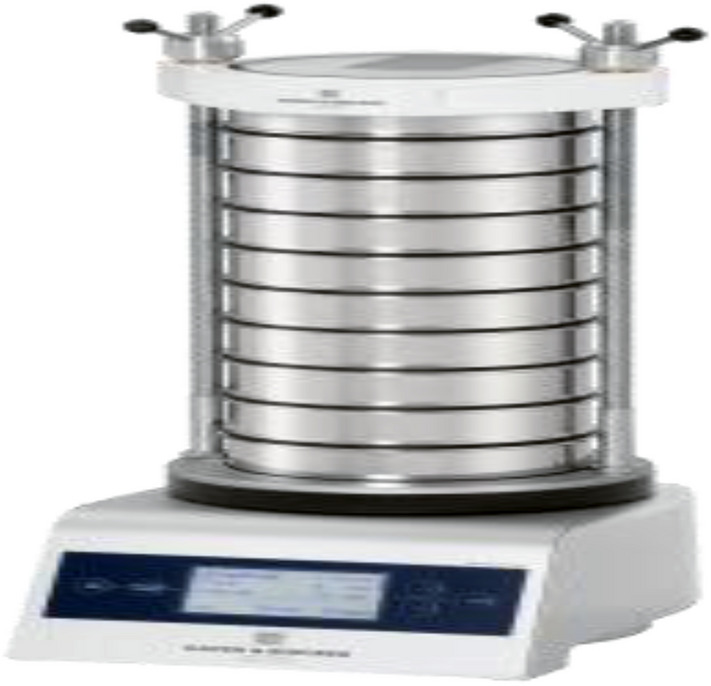
Sieve analyzer (Haver & Boecker 59302 DELDE).

The samples particle size distributions analysis was done at the Nigeria Liquefied Natural Gas (NLNG) Laboratory, University of Nigeria, Nsukka, using the digital sieve analyzer (Haver & Boecker 59302 DELDE) (Fig. 4). This was done by introducing a known weight of dried sample soil, crushed with mortar and rubber pestle, into a set of successive sieves with decreasing sieve sizes and shaken with sieve shaker for 15 min. The successive percentages of soil particles retained were calculated^32,33^. The process of soil particle analysis involved cleaning the sieve shaker's sieves, recording their weights, drying soil samples, and arranging sieves (sizes ranging from 2–0.002 mm) in ascending order. After weighing the samples, the successive percentages of soil particles retained in each pan were calculated for soil classification^34^.

### Determination of soil heavy metal concentration

Heavy metal concentrations in the sample site were analyzed in the lab to determine metal concentration levels in the study region and surroundings. This was done to determine their impact of mechanical and auto-mobile repair operations, as well as waste discharge, on soil content and quality in the study area, to classify soil suitability within mechanic villages and suggest remedy measures in maintaining environmental sustainability over the study area^35^. Moreover, implementing a robust Quality Assurance/Quality Control (QA/QC) procedure is vital for ensuring the reliability and accuracy of soil sampling and analysis. The procedure involves thorough pre-sampling planning, standardized sampling protocols, rigorous field quality control measures, careful sample handling and transport, meticulous laboratory analysis, comprehensive data validation and quality assurance checks, and detailed reporting. By adhering to these steps, researchers can minimize errors, ensure data integrity, and provide accurate insights into the spatial variability of heavy metal concentrations in soil^36^.

The dried fine soil sample was weighed and poured in a round bottom flask. Subsequently, 10 cm^3^ of concentrated nitric acid was added to the soil. The digested mixture was placed on a hot plate and heated intermittently to ensure a steady temperature of 150 °C for a period of 5 h, until a clear solution was obtained^35^. To recover any residual metal, the mixture was allowed to cool to room temperature before being filtered through Whatman No. 1 filter paper into a 50 cm^3^ volumetric flask and made up to the normal mark with de-ionized water after rinsing the reacting vessels. The filtrate was then stored in pre-cleaned polyethylene storage bottles ready for analysis. Heavy metal concentrations were determined using the Spectrophotometer at the National Center for Energy Research and Development (NCERD), Nsukka. The manufacturer's specifications were followed in setting and operating the instrument. The digested samples were analyzed for the concentrations of heavy metals (Fe, Zn, Cu, As, Cd, and Pb) using flame atomic absorption spectrophotometer (FAAS, Model: AA-320N). Final concentrations of the metals in the soil samples were calculated using Eq. (2) according to Adebayo et al*.*^37^.

2 $$concentration \left( {\frac{mg}{{kg}}} \right) = Conc. \left( \frac{mg}{L} \right) \times Conc. \left( {\frac{mg}{{kg}}} \right) = Conc. \left( \frac{mg}{L} \right) \times VW$$

where *V* = Final volume (50 ml) of solution, and *W* = Initial weight (0.5 g) of sample measured.

The result of the analysis was compared to environmental recommended standard and subjected to pollution assessment indices, to ascertain the level of heavy metal pollution in the study area.

### Assessment of auto mechanic workshop activities on the surrounding soil environment

The results of the soil analysis from the workshop clusters were compared to those obtained from the control sample points, assumed to be unpolluted or background values. Puyate et al.^38^ defined background values as the maximum level of an element in an environment beyond which the environment is said to be polluted with the element. The results obtained from the laboratory test were further subjected to statistical analysis test using SPSS Software (10.2.2). SPSS was used to determine the Range, Mean, Sum and Standard deviation of the heavy metal concentration in the sampled soils and the metals concentration was further subjected to comparison with the WHO soil standard for heavy metal level in soil^39^. Pollution indices recommended by Hakanson^40^; index of geo-accumulation (I-geo) which allows for pollution evaluation by comparing existing and pristine concentrations of contaminants calculated using Eq. (3)^41,42^.

3 $$I_{geo} = \log_2 \left( {\frac{C_n }{{1.5B_n }}} \right)$$

where Cn is the heavy metal concentration in the enriched sample and B_n_ is the metal concentration in the unpolluted (control) samples. The factor 1.5 is used to reduce the impact of potential changes in the history or control values that may be due to lithogenic variations in the soil^43^. As shown in Table 1, the degree of metal exposure is classified into seven contamination groups in order of increasing numerical value of the index^44^.

**Table 1:** Seven classes of geo-accumulation index^43^.

Class	Value of soil quality
< 0	Unpolluted
0–1	unpolluted to moderately polluted
1–2	moderately polluted
2–3	moderately polluted to highly polluted
3–4	highly polluted
4–5	highly polluted to very highly polluted
> 5	Very highly polluted

Also, Contamination factor as proposed by Hakanson^40^ is as presented in Eq. (4). where $C_{0 - 1}^i$ is the mean content of metals from at least 5 sample sites and $C_n^i$ is the pre-industrial concentration of individual metals. $C_f$ were calculated from the mean concentrations of the heavy metals in the study areas with the control sampling site taken to represent the background values. According to Akoto et al*.*^45^, $C_f$ values between 0.5 and 1.5 indicate that the metal is entirely from crust materials or natural processes; whereas $C_f$ values greater than 1.5 suggest that the sources are more likely to be anthropogenic. Hakanson^40^ reported terminology (Table 2) that may be used in risk index approach to get a uniform way of describing the contamination factor.

4 $$C_f = \frac{{C_{0 - 1}^i }}{C_n^i }$$

Moreover, Quantification of anthropogenic concentration of metals (QoC) which employs the concentration in the control samples to represent the lithogenic metal and is calculated as presented in Eq. (5). Where *x* = average concentration of the metal in the soil under investigation, and $x_c$ = average concentration of the metal in the control samples^46^.

5 $${\text{Quantification }}\;{\text{of}}\;{\text{ anthropogenic }}\;{\text{metal}} = \frac{x - x_c }{x} \times 100$$

These evaluation indices were adapted to ascertain the impact of auto mechanic activities on the concentration of toxic trace metals in soil.

**Table 2:** Terminology used in risk index for describing contamination factor^40^.

Contamination factor	Classification
C_f_ < 1	Low contamination
1 ≤ C_f_ < 3	Moderate contamination
3 ≤ C_f_ < 6	Considerate contamination
C_f_ > 6	Very high contamination

### Spatial distribution of heavy metals

The ArcGIS 10.2.2 software was used for spatial interpolation of the heavy metals and aided the spatial distribution of their contamination across the study area. The concentration movements were monitored down the soil profile from 0 to 30 cm and 30–60 cm. Their lateral movement on the soil surface was also mapped spatially.

#### Spatial interpolation methods

Two interpolation techniques were used, Spline and Kriging interpolation methods. Spline uses a deterministic approach while Kriging is geostatistical. These two methods with different approach were chosen to compare their performance. There are many interpolation methods available for use but, Inverse Distance Weighting (IDW)—a type of deterministic method for multivariate interpolation with a known scattered set of points—and Kriging were chosen because they are widely used for spatially explicit hydrologic/watershed models that require continuous data surfaces like temperature and evapotranspiration^47,48^. The IDW is a simple and intuitive deterministic interpolation method based on the principle that sample values closer to the prediction location have more influence on prediction value than sample values farther apart and its “bull's eye” effect (higher values near observed location) and edgy surface are advantages. The Kriging tool fits a mathematical function to a specified number of points, or all points within a specified radius, to determine the output value for each location^49^. The basic tool of geostatistics and Kriging is the semivariogram. It captures the spatial dependence between samples by plotting the semi variance against separation distance. In Kriging, the weights are based not only on the distance between the measured points and the prediction location, but also on the overall spatial arrangement of the measured points^50^.

### Statistical analysis

The PROC ANOVA subroutine of the SAS (2017) application was used to perform analyses of variance, and interactions for the various parameters were calculated. The Duncan's Multiple Range Test (DMRT) was used to differentiate the means of soil parameters at a 5% level of significance (P = 0.05)^51,52^. The equation is represented in Eq. (6)

6 $$s^2 = \frac{1}{n - 1}\sum (y - y_1 )^2$$

The Pearson equation given in Eq. (7) was used to calculate correlation (r) value.

7 $$r = \frac{{N \left( {\sum XY} \right) - \left( {\sum X} \right)\left( {\sum Y} \right)}}{{\sqrt {{\left[ {N\left( {\sum X^2 } \right) - \left( {\sum X} \right)^2 } \right] \left[ {N\left( {\sum Y^2 } \right) - \left( {\sum Y} \right)^2 } \right]}} }}$$

where r is the Pearson correlation coefficient, which gives a value between + 1 and 1 when measured between two variables X and Y.

The difference in metal content within the study area was examined using a simple student’s' test examination. Levene’s Student Test for Significant Difference Statistical analysis was used to determine the variation between the principal sample and the control sample in the study area, statistical analysis was performed using the Statistical Package for Social Science (SPSS Version 21)^53^. At a 95% confidence level, Levene's Independent Sample test was used to calculate the mean significant difference between the levels of accuracy of two interpolation methods. This test was also used for comparative purposes between the sample result and the control sample result. Under the null hypothesis, a t-test is any statistical hypothesis test in which the test statistic matches a student’s t-distribution. It is most often used when the test statistic will obey a normal distribution if the value of a scaling term in the test statistic was known. T-test uses means and standard deviations of two samples to make a comparison^54,55^. The equation is presented in Eq. (8):

8 $$t = \frac{{\overline{X_1 } - \overline{X_2 }}}{{s_{\overline{\vartriangle }} }}$$

where X_2_ = Mean of first set of values; X_1_ = Mean of second set of values; S_1_ = Standard deviation of first set of values; S_2_ = Standard deviation of second set of values; n_1_ = Total number of values in first set; n_2_ = Total number of values in second set and $s_{\overline{\vartriangle }} = \sqrt {{\frac{s_1^2 }{{n_1 }} + \frac{s_2^2 }{{n_2 }}}}$.

To compare different methods, the accuracy of each of them was evaluated. Cross validation was useful for this purpose; it permitted the researcher to find the accuracy level of predictive values by distinguishing between the training set and the validation set—the first used for model generation, the second for model evaluation^56^. Different approaches are usually adopted for cross validation. Leave-one-out method is based on the removal of a point from the data to be interpolated, the use of the other points to estimate a value at the location of the removed point, and the performance test by means of the removed data. The difference between the known value and estimated value in each removed point, is calculated to evaluate the performance of the assumed interpolation method^57,58^.

### Consent to participate

All authors were highly cooperative and involved in research activities and preparation of this article.

## Results and discussion

### Geospatial location of sample points

The results of the various coordinate values collected from the sampled points within the study area using the GPS Device (Germin Etrex 30) is presented in Table 3. The spatial map of the study area produced with the coordinate points is as shown in Fig. 3 and was produced using the ArcGIS software 10.2.2.

**Table 3:** Location of sampling points in the mechanic village.

Stations	Latitude (°N)	Longitude (°E)	Altitude (m)
1	6.83986	7.38089	452
2	6.84175	7.38044	448
3	6.84153	7.37853	457
4	6.84228	7.37656	449
5	6.84053	7.37667	453
6	6.83989	7.37697	448
7	6.83772	7.37683	455
8	6.83897	7.37867	452
9	6.83981	7.37878	452
10	6.84081	7.37886	459
11	6.84078	7.3795	444
12	6.84011	7.38139	451
13	6.84247	7.38067	453
14	6.84208	7.38217	467
15	6.84106	7.38186	452
16	6.83878	7.38103	450
17	6.83922	7.38244	455
18	6.83758	7.37214	432
19	6.83736	7.37022	442
20	6.84331	7.37492	448

### Soil texture and moisture analysis

The particle size distribution and moisture content of the soil samples taken at 0–30 cm depth within the workshop cluster gave a textural class of sandy clay loam, sandy loam, and moisture content 6.08 to 13.92%. Analysis of particle size distribution of soil samples at 0–30 cm depth within the cluster gave a high sand content with percentage of sand ranging between 72 to 82%; clay content ranging from 13 to 21% and silt ranging from 3 to 5% as shown in Table 4. The textural classes for the locations ranged from sandy loam to sandy clay loam. The high proportion of sand in the soil sample can be attributed to the parent materials, which are derived from coastal plain sands of southern Nigeria^59,60^. The result of the particle size analysis and moisture content for the control (outside the workshop cluster) at 0–30 cm depth and shows a textural class of sandy loam and moisture content which ranges from 6.94 to 16.71% (see Table 4). The particle size distribution results of soil samples at 0–30 cm depth outside the cluster showed that sand has a greater content with 72–84%, silt content ranges from 5–9% and clay content has 11–21%. Generally, the soil outside the cluster exhibits a diverse particle size distribution, with varying proportions of sand, silt, and clay. The high sand content suggests relatively good drainage, while the substantial clay content indicates high water retention capacity. The substantial sand content indicates a coarse soil texture, potentially impacting its ability to retain nutrients. Conversely, the presence of silt contributes to a smoother texture and enhances the soil's nutrient retention capacity^61,62^.

**Table 4:** Result of soil particle size and moisture content analysis from depth of 0–30 cm within and outside the mechanic village cluster.

Sampling points	Moisture content (%)	Particle size (%)			Textural class
		Clay	Silt	Sand	
STN 1	9.53	23	5	72	Sandy clay loam
STN 2	8.99	21	7	72	Sandy clay loam
STN 3	9.21	17	7	76	Sandy loam
STN 4	8.04	21	7	72	Sandy clay loam
STN 5	6.46	17	7	76	sandy loam
STN 6	13.92	19	3	78	Sandy loam
STN 7	8.19	13	5	82	Sandy loam
STN 8	9.41	15	5	80	Sandy loam
STN 9	6.08	15	7	78	Sandy loam
STN 10	11.06	21	7	72	Sandy clay loam
Control (outside the cluster)					
STN 11	16.71	15	9	76	Sandy loam
STN 12	11.57	17	5	78	Sandy loam
STN 13	12.17	17	7	76	Sandy loam
STN 14	9.78	21	7	72	Sandy clay loam
STN 15	12.04	13	5	82	Sandy loam
STN 16	6.94	11	5	84	Loam sand
STN 17	13.02	17	5	78	Sandy loam
STN 18	11.78	19	7	74	Sandy loam
STN 19	7.48	17	5	78	Sandy loam
STN 20	12.82	19	7	74	Sandy loam

The results of the particle size analysis and moisture content for the depth of 30 to 60 cm within and outside the workshop cluster are as presented in Table 5. The obtained results show a textural class of sandy loam and sandy clay loam with moisture content which ranges from 7.28 to 14%. Moreover, the result of the particle size analysis and moisture content for the control (outside the workshop cluster) at 30 to 60 cm depth and shows a textural class of sandy loam, loam sand and sandy clay loam and moisture content which ranges from 5.26 to 14.49%. Overall, it was observed that for the moisture content results obtained within the cluster is less than the result for soil obtained outside the cluster^63,64^.

**Table 5:** Result of soil particle size and moisture content analysis from depth of 30–60 cm within and outside the mechanic village cluster.

Sampling points	Moisture content (%)	Particle size (%)			Textural class
		Clay	Silt	Sand	
STN 1	12.69	21	5	74	Sandy clay loam
STN 2	10.88	21	5	74	Sandy clay loam
STN 3	10.35	19	7	74	Sandy loam
STN 4	11.89	19	5	76	Sandy loam
STN 5	7.28	17	9	72	Sandy loam
STN 6	11.01	19	5	76	Sandy loam
STN 7	11.04	13	5	82	Sandy loam
STN 8	10.66	13	7	80	Sandy loam
STN 9	13.42	15	7	78	Sandy loam
STN 10	14.00	21	5	74	Sandy clay loam
Control (outside the cluster)					
STN 11	9.11	13	7	80	Sandy loam
STN 12	10.81	17	7	76	Sandy loam
STN 13	11.3	19	7	74	Sandy loam
STN 14	14.26	21	7	72	Sandy clay loam
STN 15	14.49	17	3	80	Sandy loam
STN 16	5.26	9	5	86	Loam sand
STN 17	7.36	19	3	78	Sandy loam
STN 18	10.34	19	7	74	Sandy loam
STN 19	12.91	19	5	76	Sandy loam
STN 20	12.41	19	7	74	Sandy loam

### Concentration levels of heavy metals

Data regarding heavy metal content were obtained through laboratory testing of samples collected from depths ranging from 0 to 30 cm and 30 to 60 cm. The resulting values are detailed in Tables 6 and 7 for both the cluster and control areas. Subsequently, these data were aggregated and summarized to calculate means and standard deviations. The analyzed values were then compared with those reported in other studies^65,66^.

**Table 6:** Heavy metal concentration for soil from depths of 0–30 cm within the cluster (mg/kg).

Sample	Cu	Fe	Zn	Pb	As	Cd
STN 1	0.794	3.685	18.892	1.137	2.862	0.225
STN 2	0.878	2.576	19.811	1.225	2.443	0.281
STN 3	0.687	4.671	19.947	1.282	3.062	0.397
STN 4	0.742	2.598	27.222	0.881	1.886	0.246
STN 5	1.107	4.883	22.559	1.124	2.338	0.271
STN 6	0.668	3.794	27.648	1.145	3.894	0.154
STN 7	0.616	2.469	21.676	1.126	3.552	0.378
STN 8	0.574	2.862	20.813	1.225	2.491	0.187
STN 9	0.696	2.977	27.826	1.138	4.125	1.258
STN 10	0.749	2.758	28.248	0.865	2.467	0.249
Average	0.751	3.327	23.464	1.115	2.912	0.365
Std. Dev	0.152	0.886	3.818	0.138	0.734	0.323
Outside cluster (control) (mg/kg)						
STN 11	0.713	2.973	10.641	0.932	1.746	0.068
STN 12	0.721	2.914	13.323	0.603	2.627	0.119
STN 13	0.657	2.356	12.332	1.046	2.016	0.105
STN 14	0.585	2.402	16.776	1.005	2.528	0.102
STN 15	0.609	2.368	11.317	1.219	1.754	0.057
STN 16	0.702	3.645	12.84	1.091	2.222	0.071
STN 17	0.764	1.551	14.56	1.104	1.125	0.108
STN 18	0.473	1.947	13.428	0.567	1.631	0.113
STN 19	0.526	2.355	10.837	0.698	1.293	0.124
STN 20	0.428	2.644	12.353	0.765	2.166	0.106
Average	0.618	2.516	12.841	0.903	1.911	0.097
Std. Dev	0.114	0.577	1.847	0.229	0.494	0.023

**Table 7:** Heavy metal concentration for soil samples from depths of 30–60 cm within the cluster (mg/kg).

Sample	Cu	Fe	Zn	Pb	As	Cd
STN 1	0.742	2.454	33.852	1.266	2.832	0.125
STN 2	0.831	1.772	23.474	0.975	2.118	0.124
STN 3	0.718	2.683	28.858	1.284	2.467	0.148
STN 4	0.709	2.642	31.347	0.873	1.749	0.153
STN 5	0.784	3.437	32.986	1.362	2.247	0.161
STN 6	0.597	2.776	29.369	1.265	3.17	0.137
STN 7	0.656	3.785	31.875	1.271	3.176	0.135
STN 8	0.643	3.368	28.691	1.287	2.296	0.126
STN 9	0.747	2.929	32.143	1.199	3.292	0.104
STN 10	0.734	3.967	28.537	1.318	2.648	0.112
Average	0.716	2.981	30.113	1.21	2.600	0.133
SD	0.069	0.664	3.013	0.158	0.515	0.018
Outside cluster (control) (mg/kg)						
STN 11	0.706	3.044	15.922	0.664	1.793	0.108
STN 12	0.683	2.472	12.731	1.036	1.334	0.012
STN 13	0.701	2.048	14.454	1.117	1.727	0.117
STN 14	0.567	2.221	16.217	0.835	1.366	0.104
STN 15	0.591	1.633	17.528	1.071	2.265	0.111
STN 16	0.747	2.101	16.865	1.082	2.077	0.102
STN 17	0.715	1.857	17.933	1.101	1.226	0.125
STN 18	0.466	1.991	15.216	0.672	1.667	0.114
STN 19	0.506	2.718	15.754	0.753	1.276	0.103
STN 20	0.432	2.248	14.739	0.537	1.957	0.014
Average	0.611	2.233	15.736	0.887	1.669	0.091
SD	0.115	0.417	1.546	0.219	0.362	0.042

Examination of soil sample results obtained from depths of 0–30 cm and 30–60 cm revealed a dispersion and accumulation of trace metals around ten points within the cluster. The average concentrations of the heavy metals were noted to decline progressively with depth, while Zinc (Zn) and Lead (Pb) exhibited an upward trend. This observation aligns with the results reported in Ololade^5^, which indicated a general decrease in average concentrations of Cadmium (Cd), Chromium (Cr), Copper (Cu), Iron (Fe), and Nickel (Ni) with increasing soil depth, while Lead (Pb) and Zinc (Zn) concentrations showed an upward trend. Also, it was noted that the levels of heavy metals within the auto mechanic clusters exceeded those observed outside the clusters (control). The investigation into vertical variations of heavy metal concentrations in soil at depths of 0–30 cm and 30–60 cm highlights potential trends in heavy metal contamination. The elevated concentrations of certain metals may be attributed to the presence of additives, consisting of metals in various proportions, found in lubricants used by auto mechanics^67^.

The automobile clusters exhibited relatively low copper (Cu) concentrations at both depths (0–30 cm & 30–60 cm), with mean values of 0.751 mg/kg and 0.716 mg/kg, respectively. These concentrations were significantly above the mean control value. The presence of copper at this level could be attributed to electrical components, such as wires, as well as waste oil and alloys from corroded vehicle scraps, which have accumulated in the vicinity of these clusters over an extended period. The gradual leaching of metals from the corrosion process may have contributed to the copper content observed in the soil^13^.

The average concentration of zinc in the soil was found to be 23.464 mg/kg within the clusters at depths of 0–30 cm, compared to 12.84 mg/kg outside the clusters. Similarly, at depths of 30–60 cm, a higher concentration of 30.113 mg/kg was observed within the clusters compared to 12.84 mg/kg outside the clusters^68^. These findings suggest anthropogenic contamination in the soil samples, with elevated levels of zinc particularly notable. The increased zinc content in automotive clusters can be attributed to its presence in various lubricating oil additives commonly used in automotive maintenance activities^66^. Zinc-based additives, such as zinc dialkyldithiophosphate (ZDDP), are frequently employed to enhance lubrication and reduce wear in engine components. Over time, these additives can accumulate in the soil through deposition from vehicle emissions, spillage during maintenance activities, or leaching from discarded lubricants^69,70^. However, it is noteworthy that the zinc concentration observed in this study surpassed that of the six other metals analyzed. This may be due to the widespread use of zinc-based additives in automotive lubricants, coupled with the high frequency of maintenance activities in auto-mechanic workshop clusters^71^.

Cadmium (Cd) concentrations of 0.365 mg/kg was obtained at 0–30 cm depth and 0.133 mg/kg was derived at 30–60 cm depth. According to Jarup^72^ and Ebong et al.^73^, the presence of cadmium in automotive clusters may be attributed to nickel–cadmium batteries, motor oil, and disposal sludge. Dabkowska–Naskret^74^ and Morka et al.^75^ also noted that lubricating oils, vehicle wheels, and metal alloys used for engine part hardening could contribute to cadmium contamination.

The concentrations of lead (Pb) were found to increase with depth, measuring 1.115 mg/kg at 0–30 cm and 1.21 mg/kg at 30–60 cm depths, slightly higher than control values. Lead naturally occurs in soils with concentrations typically ranging from 1 to 200 mg/kg, averaging 15 mg/kg. The elevated concentrations with depth may result from processes such as leaching, deposition, and historical anthropogenic activities^76^. Factors influencing lead mobility include soil pH, organic matter, and mineral composition. These activities can result in the deposition of lead-containing dust and residues, which may persist in the soil and gradually migrate downwards over time^77^.

The mean iron (Fe) levels in the studied clusters were 3.327 mg/kg at a depth of 0–30 cm and 2.981 mg/kg at a depth of 30–60 cm, respectively. These values were significantly lower compared to those reported by Morka et al.^75^, which were 64.45 mg/kg and 92.11 mg/kg, respectively, at similar depths. The presence of iron observed in this study can be explained by the fact that natural soils inherently contain substantial concentrations of iron. Furthermore, the presence of iron can also be attributed to wear on automobile crankshafts and damage to vehicle bodies^78,79^.

Arsenic (As) levels in the soils showed mean values of 2.912 mg/kg at a depth of 0–30 cm and 2.648 mg/kg at a depth of 30–60 cm. These values suggest anthropogenic contributions to the soil samples. Generally, the relative abundance of the six metals at a depth of 0–30 cm and 30–60 cm was as follows: Zn > Fe > As > Pb > Cu > Cd. The results also indicated a widespread distribution of the metals across the two profile layers. The widespread distribution of these metals across both soil layers underscores the complexity of their sources and transport mechanisms^80^. Factors such as soil pH, organic matter content, and soil texture can influence the mobility and retention of metals in soil. Additionally, climatic conditions, land use practices, and historical land management practices can further influence metal distribution patterns^81^.

### Statistical analysis of heavy metals’ concentration levels

To compare heavy metals' concentration levels between samples within and outside the clusters (control), an analysis of variance (ANOVA) statistical is conducted for soil obtained from 0–30 cm and 30–60 cm depth. This involves collecting data from soil samples within and outside the clusters, defining two groups, formulating hypotheses, selecting the appropriate ANOVA model, calculating the F-statistic and p-value, and interpreting the results^82,83^. This analysis helps determine if there's a significant difference in heavy metals' concentrations between the two sample groups, providing valuable insights for environmental monitoring and management as shown in Tables 8 and 9. The statistical analysis conducted using Microsoft Excel software revealed a significant difference in heavy metal concentrations between samples collected within and outside the auto mechanic cluster in the study area with p-value < 0.05. This disparity suggests that activities associated with auto mechanics within the cluster significantly influence heavy metal levels compared to areas outside the cluster^84,85^.

**Table 8:** ANOVA for metal concentration in soils within and outside the cluster at 0–30 cm depth.

Source of variances	Heavy metals	DF	MS	SS	F	*P* value	Fcrit	Remarks
Between Groups	Cu	1	0.089	0.089	4.926	0.040	4.414	F > Fcrit (SD)
Within Groups		18	0.018	0.325				
Between Groups	Fe	1	3.295	3.295	5.891	0.026	4.414	F > Fcrit (SD)
Within Groups		18	0.559	10.067				
Between Groups	Zn	1	564.294	564.294	62.740	0.000	4.414	F > Fcrit (SD)
Within Groups		18	8.994	161.895				
Between Groups	Pb	1	0.224	0.224	6.282	0.022	4.414	F > Fcrit (SD)
Within Groups		18	0.036	0.643				
Between Groups	As	1	5.012	5.012	12.808	0.002	4.414	F > Fcrit (SD)
Within Groups		18	0.391	7.044				
Between Groups	Cd	1	0.357	0.357	6.820	0.018	4.414	F > Fcrit (SD)
Within Groups		18	0.052	0.943				

DF: Degrees of Freedom, SS: Sum-of-squares, MS: Mean squares, SD: Significant difference.

**Table 9:** ANOVA for metal concentration in soils within and outside the cluster at 30–60 cm depth.

Source of variances	Heavy metals	DF	MS	SS	F	*P* value	Fcrit	Remarks
Between Groups	Cu	1	0.055	0.055	6.128	0.023	4.414	F > Fcrit (SD)
Within Groups		18	0.009	0.161				
Between Groups	Fe	1	2.798	2.798	9.112	0.007	4.414	F > Fcrit (SD)
Within Groups		18	0.307	5.526				
Between Groups	Zn	1	1033.534	1033.534	180.250	0.000	4.414	F > Fcrit (SD)
Within Groups		18	5.734	103.210				
Between Groups	Pb	1	0.522	0.522	14.312	0.001	4.414	F > Fcrit (SD)
Within Groups		18	0.036	0.657				
Between Groups	As	1	4.331	4.331	21.892	0.0002	4.414	F > Fcrit (SD)
Within Groups		18	0.198	3.561				
Between Groups	Cd	1	0.009	0.009	8.353	0.010	4.414	F > Fcrit (SD)
Within Groups		18	0.001	0.019				

DF: Degrees of Freedom, SS: Sum-of-squares, MS: Mean squares, SD: Significant difference.

Several factors contribute to these observed differences. Within the auto mechanic cluster, intensive vehicular maintenance and repair activities generate metal particles and dust from metal-based automotive parts and machinery. Additionally, the handling and disposal of automotive fluids containing heavy metals pose contamination risks, particularly if waste management practices are inadequate^86^.

Furthermore, infrastructure within the cluster, such as workshops and waste disposal sites, may exacerbate heavy metal contamination due to improper waste management practices. On the contrary, areas outside the cluster generally experience lower heavy metal levels due to reduced exposure to automotive-related activities. While some background levels of heavy metals may still be present in the environment due to natural sources or other anthropogenic activities, they are likely to be lower compared to concentrations found within the cluster^87^. Overall, addressing heavy metal pollution within auto mechanic clusters is essential for mitigating potential risks to human health and the environment. Implementing pollution control measures, promoting proper waste management practices, and conducting regular monitoring are crucial steps toward ensuring environmental sustainability in such areas^88^.

### Soil pollution assessment of nsukka auto mechanic cluster

The result of the mean of each studied heavy metal at the different studied depths was compared with the WHO’s standard as shown in Table 10a, b^89^. The presented results for Cu, Fe, Zn, Pb, As and Cd were observed to be within the FAO/WHO recommended standard for both studied depths. However, the metal concentration levels generally reduced as the depth increases. Hence the impact of the activities on the studied site may be termed minimal. This may be attributed to the other uses of the spent oils in the vicinity^90^.

**Table 10:** (a) Heavy metal concentration (mg/kg) of soil from 0 to 30 cm against WHO standard. (b) Heavy metal concentration (mg/kg) of soil from 30 to 60 cm against WHO standard.

Parameter	Cu	Fe	Zn	Pb		As	Cd
*(a)*							
No. of samples	10	10	10	10		10	10
Range	0.533	2.414	9.356	0.417		2.239	1.104
Minimum	0.574	2.469	18.892	0.865		1.886	0.154
Maximum	1.107	4.883	28.248	1.282		4.125	1.258
Sum	7.511	33.273	234.642	11.148		29.12	3.646
Mean	0.7511	3.3273	23.4642	1.1148		2.912	0.3646
Std. error	0.048	0.280	1.207	0.044		0.232	0.102
Std. deviation	0.152	0.886	3.818	0.138		0.734	0.323
Variance	0.023	0.785	14.578	0.019		0.539	0.104
WHO Standard	100	400	300	50		20	3
*(b)*							
No. of samples	10	10	10	10		10	10
Range	0.234	2.195	10.378	0.489		1.543	0.057
Minimum	0.597	1.772	23.474	0.873		1.749	0.104
Maximum	0.831	3.967	33.852	1.362		3.292	0.161
Sum	7.161	29.813	301.132	12.1		25.995	1.325
Mean	0.7161	2.9813	30.1132	1.21		2.5995	0.1325
Std. Error	0.022	0.210	0.953	0.050		0.163	0.006
Std. Deviation	0.069	0.664	3.013	0.158		0.515	0.018
Variance	0.005	0.440	9.077	0.025		0.265	0.0003
WHO Standard	100	400	300	50		20	3

Furthermore, the results of pollution indices; index of geo-accumulation (Igeo), Contamination factor (Cf), and anthropogenic concentration of metal (QoC), used to ascertain the pollution status of the studied environment is presented in Table 11. The results indicate that contamination index values falling between 0.5 and 1.5 suggest a predominance of natural processes contributing to the presence of metals in the soil. Conversely, contamination index values exceeding 1.5 indicate a substantial influence from anthropogenic sources. Among the metals analyzed, Arsenic, Cadmium, and Zinc exhibited the highest contamination factors, with contamination factors (Cf) of 1.524, 3.759, and 1.827, respectively. These values signify a significant level of contamination by these metals in the soil, likely stemming from anthropogenic activities^91^. In contrast, Copper, Lead, and Iron displayed varying degrees of contamination, ranging from minimal to considerable, with contamination factors of 1.215, 1.235, and 1.322, respectively. However, at a depth of 30–60 cm, moderate contamination indices were observed for all the metals analyzed. Notably, contamination factors exceeding 1.5 for a metal indicate a substantial contribution from anthropogenic sources, emphasizing the potential environmental impact of human activities on heavy metal contamination in soil^92^.

**Table 11:** Pollution Indices results.

	Cu	Fe	Zn	Pb	As	Cd
Cf (0–30 cm)	1.215	1.322	1.827	1.235	1.524	3.759
Cf class	MC	MC	MC	MC	MC	CC
Cf (30–60 cm)	1.172	1.335	1.914	1.364	1.558	1.456
Cf class	MC	MC	MC	MC	MC	MC
Igeo (0–30 cm)	− 0.304	− 0.182	0.285	− 0.281	0.023	1.325
Igeo class	Unpolluted	Unpolluted	Unpolluted	Unpolluted	Unpolluted	MP
Igeo (30–60 cm)	− 0.356	− 0.168	0.351	− 0.137	0.054	− 0.043
Igeo class	Unpolluted	Unpolluted	Unpolluted	Unpolluted	Unpolluted	Unpolluted
QoC (%) (0–30 cm)	17.72068	24.38313	45.27408	18.99892	34.375	73.3955
QoC (%) (30–60 cm)	14.67672	25.09979	47.74385	26.69421	35.79535	31.32075

MC: Moderate contamination, CC: Considerate contamination, MP: moderately polluted.

The Geo-accumulation Index (Igeo) values for soil samples collected at depths of 0–30 cm and 30–60 cm were calculated to assess heavy metal contamination levels. At 0–30 cm, Copper, Lead, and Iron showed uncontaminated levels, while Zinc and Arsenic exhibited near-background contamination levels. Cadmium was moderately polluted. At 30–60 cm, all metals analyzed showed predominantly uncontaminated levels. Overall, the results suggest that the soil samples, particularly those collected at greater depths, generally exhibited low to minimal levels of contamination for the metals analyzed. This indicates a relatively low environmental impact from anthropogenic sources, at least in terms of heavy metal contamination, in the study area. Further investigation is warranted, particularly for the moderate pollution of Cadmium in surface soil layers^93^.

The Quality Index (QoC) values for the analyzed heavy metals were positive for both depths of 0–30 cm and 30–60 cm, suggesting that heavy metal contamination primarily originated from anthropogenic sources. In terms of heavy metal contamination order, soil samples obtained at 0–30 cm depth showed the highest contamination for Cadmium (Cd), followed by Zinc (Zn), Arsenic (As), Iron (Fe), Lead (Pb), and Copper (Cu). On the other hand, for soil collected at 30–60 cm depth, the order of heavy metal contamination was led by Zinc (Zn), followed by Cadmium (Cd), Arsenic (As), Lead (Pb), Iron (Fe), and Copper (Cu). These findings indicate variations in heavy metal contamination levels between the two soil depths, with different metals exhibiting varying degrees of contamination. The positive QoC values reaffirm the predominantly anthropogenic nature of heavy metal contamination in the soil, emphasizing the importance of further investigation and remediation efforts to mitigate potential environmental and health risks^94^.

### Spatial variability of heavy metal concentration in the mechanic village

**Figure 5:**
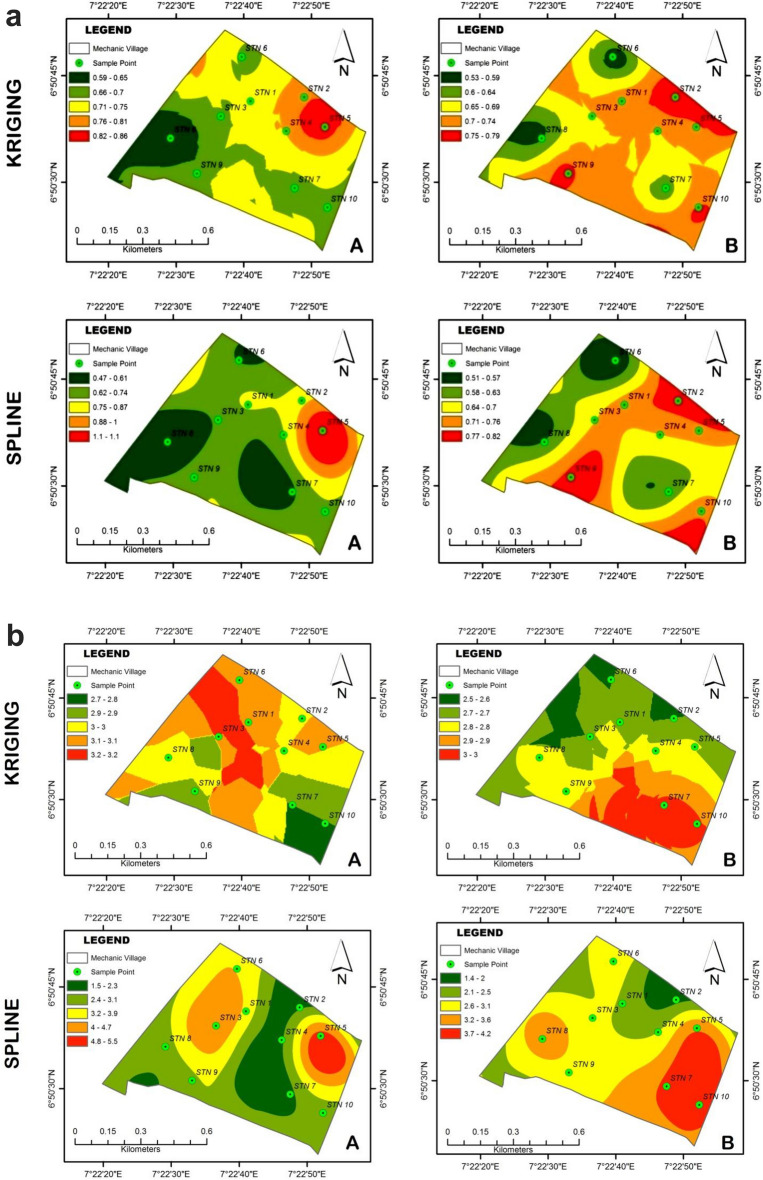

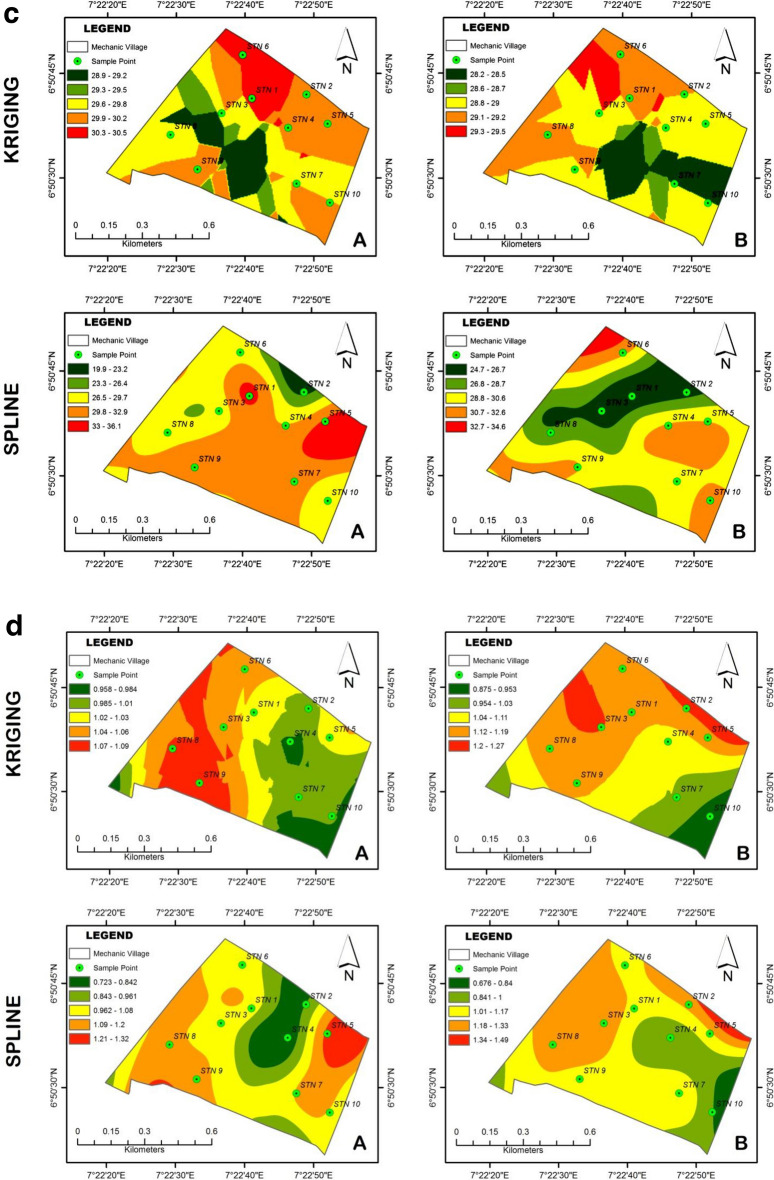

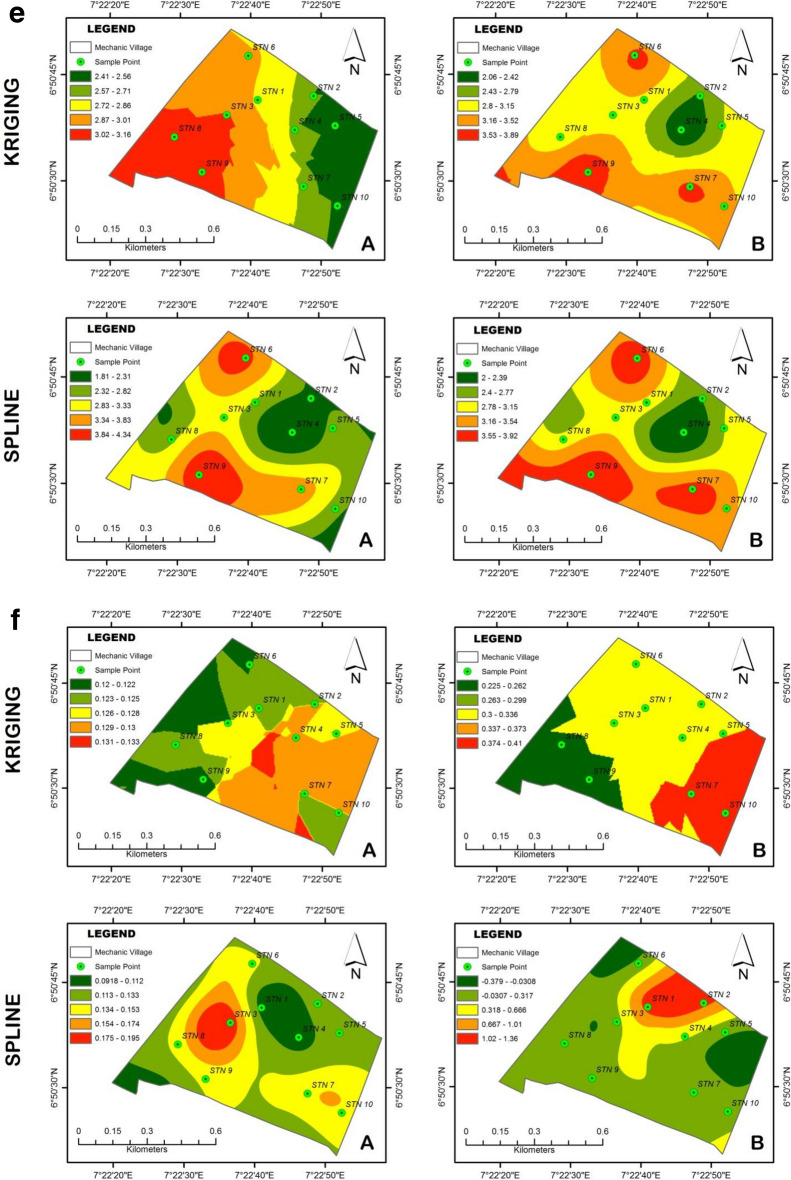
(**a**) Interpolation distribution of Kriging and Spline for Cu: A = 0–30 cm; B = 30–60 cm. (**b**) Interpolation distribution of Kriging and Spline for Fe: A = 0–30 cm; B = 30–60 cm. (**c**) Interpolation distribution of Kriging and Spline for Zn: A = 0–30 cm; B = 30–60 cm. (**d**) Interpolation distribution of Kriging and Spline for Pb: A = 0–30 cm; B = 30–60 cm. (**e**) Interpolation distribution of Kriging and Spline for As: A = 0–30 cm; B = 30–60 cm. (**f**) Interpolation distribution of Kriging and Spline for Cd: A = 0–30 cm; B = 30–60 cm.

The spatial distribution of the various heavy metal for the two depths as determined using the two interpolation methods are shown in Fig. 5a–f generated with the aid of ArcGIS Software. Statistical parameters of all residuals were calculated to facilitate the comparison of different approaches. The Interpolation results were subjected to accuracy assessment to determine the best interpolation method that best predicts the pattern of heavy metal distribution over the study area. The accuracy of each of them was evaluated using the Levene’s test of mean difference^95^. The results of the comparison are shown in Table 12a–f.

**Table 12:** (a) Accuracy comparison of kriging and spline interpolation method for (a) Cu, (b) Fe, (c) Zn, (d) Pb, (e) As, (f) Cd metal.

In between test	F	Sig	t	Df	Sig. (2-tailed)
(a)					
Kriging Value and Sample Test Data (0–30 m)	0.001	0.981	0.093	18	0.927
Spline Value and Sample Test Data Value (0–30 m)	0.165	0.689	0.131	18	0.897
Kriging Value and Sample Test Data (30–60 m)	0.165	0.689	0.131	18	0.897
Spline Value and Sample Test Data Value (30–60 m)	0	0.983	0.102	18	0.92
(b)					
Kriging Value and Sample Test Data (0–30 m)	27.878	0	− 0.573	18	0.574
Spline Value and Sample Test Data Value (0–30 m)	0.005	0.943	0.117	18	0.908
Kriging Value and Sample Test Data (30–60 m)	13.987	0.001	− 0.957	18	0.351
Spline Value and Sample Test Data Value (30–60 m)	0.023	0.88	0.036	18	0.972
(c)					
Kriging Value and Sample Test Data (0–30 m)	18.877	0	0.566	18	0.578
Spline Value and Sample Test Data Value (0–30 m)	0.001	0.975	0.093	18	0.927
Kriging Value and Sample Test Data (30–60 m)	71.332	0	0.159	18	0.875
Spline Value and Sample Test Data Value (30–60 m)	0.026	0.874	0.055	18	0.957
(d)					
Kriging Value and Sample Test Data (0–30 m)	4.926	0.04	− 0.135	18	0.894
Spline Value and Sample Test Data Value (0–30 m)	0.023	0.88	0.089	18	0.93
Kriging Value and Sample Test Data (30–60 m)	0.698	0.415	0.046	18	0.964
Spline Value and Sample Test Data Value (30–60 m)	0	0.99	− 0.017	18	0.987
(e)					
Kriging Value and Sample Test Data (0–30 m)	10.991	0.004	− 0.432	18	0.671
Spline Value and Sample Test Data Value (0–30 m)	0	0.994	0.084	18	0.934
Kriging Value and Sample Test Data (30–60 m)	0.163	0.691	0.01	18	0.992
Spline Value and Sample Test Data Value (30–60 m)	0.02	0.89	0.049	18	0.962
(f)					
Kriging Value and Sample Test Data (0–30 m)	8.882	0.008	− 1.19	18	0.25
Spline Value and Sample Test Data Value (0–30 m)	0.005	0.946	0.1	18	0.921
Kriging Value and Sample Test Data (30–60 m)	11.876	0.003	− 0.312	18	0.759
Spline Value and Sample Test Data Value (30–60 m)	0.001	0.972	− 0.089	18	0.93

The results obtained from the two interpolation methods were respectively compared with the original values for significant difference. The result of the comparison is shown in Tables 13 and 14 for the depths of 0–30 cm and 30–60 cm, respectively. From the result on Table 13 for sample collected at the depth of 0–30 cm, Spline showed a 100% strong significant variation and difference from the control (original) value for all the heavy metals tested and this therefore disqualifies the validity of the representation. Therefore, we reject the H_O_ and accept H_I_, stating that there is significant difference between the Spline interpolation result and Control data. This means that Spline interpolation method is not the best method for predicting impact of mechanic activities on the contamination of soil by heavy metal in the study area^96^.

**Table 13:** Table of significant values of interpolation tests (kriging and spline) 0–30 cm.

Variables	Kriging A	Sig	Spline A	Sig
As	0.004	Not significant	0.994	Significant
Cd	0.008	Not significant	0.946	Significant
Cu	0.110	Not significant	0.981	Significant
Fe	0.000	Not significant	0.943	Significant
Pb	0.040	Not significant	0.880	Significant
Zn	0.000	Not significant	0.975	Significant

**Table 14:** Table of significant values of interpolation tests (kriging and spline) 30–60 cm.

Variables	Kriging B	Sig	Spline B	Sig
As	0.691	Significant	0.890	Significant
Cd	0.003	Not significant	0.972	Significant
Cu	0.689	Significant	0.983	Significant
Fe	0.001	Not significant	0.880	Significant
Pb	0.415	Not significant	0.990	Significant
Zn	0.000	Not significant	0.874	Significant

Kriging on the other hand showed non-significant amongst heavy metals test and at 100% and therefore we Accept the Ho stating that there is no significant difference in the Kriging Interpolation result and the control (original) values. This means that Kriging is a better interpolation method for predicting impact of mechanic and automobile repair activities on soil contamination by heavy metals in the study area^97^.

From Table 14 for samples collected at the depth of 30–60 cm, Spline showed a 100% strong significant variation and difference from the control value in all heavy metals tested and this therefore disqualifies the validity of the representation. Therefore, we reject the H_O_ and accept H_I_, stating that there is significant difference between the Spline interpolation result and Control data. This means that Spline interpolation method is not the best method for predicting impact of mechanic activities on the soil metal properties of the study area^96,98^.

Kriging on the other hand showed non-significance amongst some of the heavy metals, including Cd, Fe, Pb, Zn and significant for As and Cu. Therefore 67% of the prediction was non-significant while 33% was significantly different. Therefore, we accept the H_o_ stating that there is no significant difference in the Kriging Interpolation result and Control (original) values. This means that Kriging is a better interpolation method for predicting impact mechanic and auto-mobile repair activities on the soil metal properties of the study area. Following our analysis, the capability of Kriging methods to adapt better than others to interpret and shape the variability of a territory is confirmed by our study^99^.

### Spatial map of elevation of the study area

**Figure 6:**
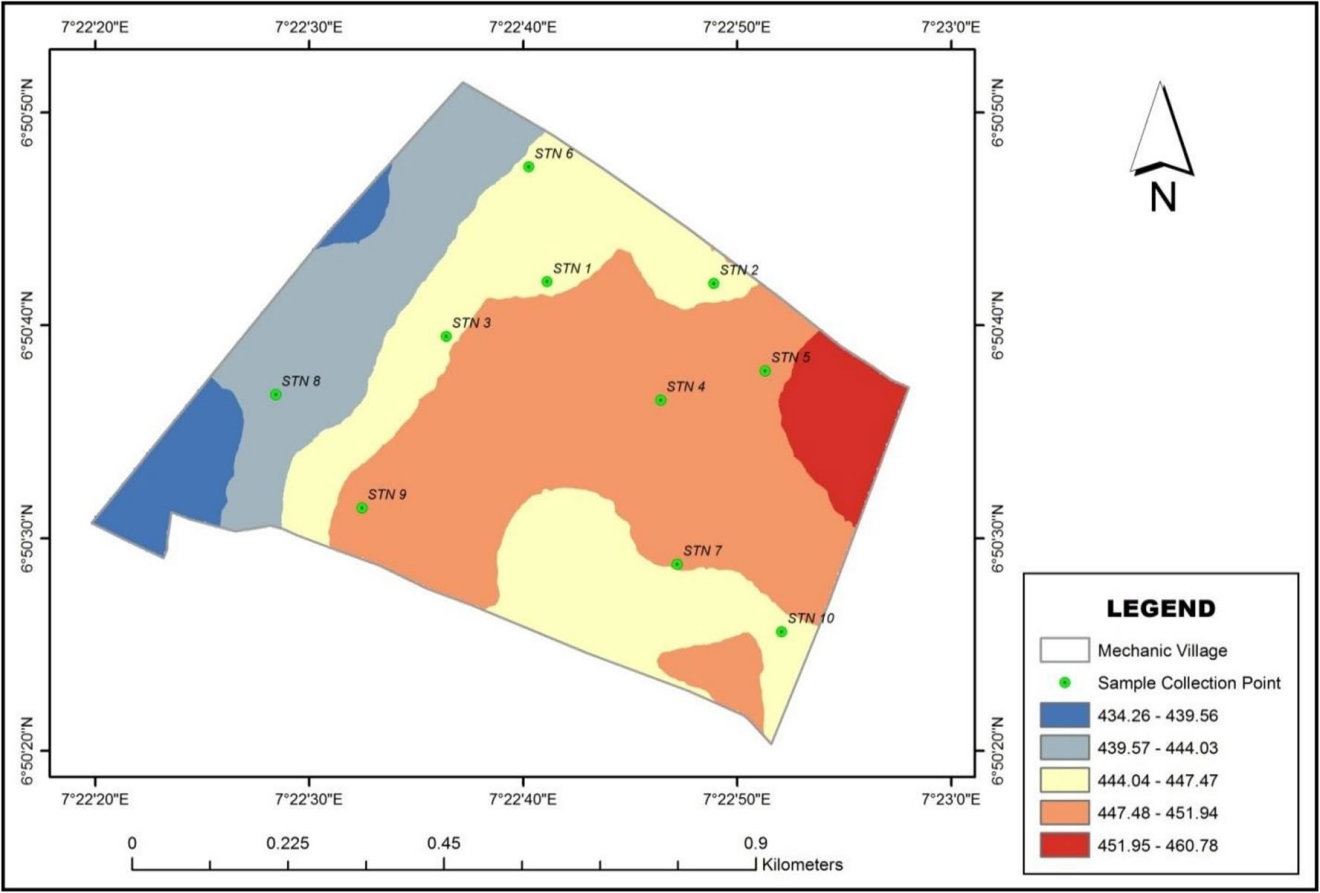
Digital elevation map of mechanic village.

Digital Elevation Model (DEM) of the study site was generated using the ArcGIS software and elevation data downloaded from United States Geology Surveys (USGS) database. The result of the DEM was subjected to test for relationship using Pearson Product Moment Correlation method. The DEM Map of the study area is presented in Fig. 6. Correlation analysis was applied using the SPSS application; this was to identify the gap between the topography of the study area and the movement, sedimentation, and infiltration of heavy metals along the area. Result of the analysis for sampled soil within of 0–30 cm and 30–60 cm are shown in Table 15a, b, respectively.

**Table 15:** Correlation result of elevation and heavy metal relationship at (a) 0–30 cm depth, (b) 30–60 cm depth.

		Elevation	Cu 0–30 cm	Fe 0–30 cm	Zn 0–30 cm	Pb 0–30 cm	As 0–30 cm	Cd 0–30 cm
(a)								
Elevation	Pearson Corr	1	− 0.085	0.173	0.145	0.525	0.04	0.658*
	Sig. (2-tailed)		0.816	0.634	0.69	0.119	0.912	0.038
No. of samples	N	10	10	10	10	10	10	10

		Elevation	Cu 30–60 cm	Fe 30–60 cm	Zn 30–60 cm	Pb 30–60 cm	As 30–60 cm	Cd 30–60 cm
(b)								
Elevation	Pearson Corr	1	0.159	0.663*	0.055	− 0.163	0.313	− 0.398
	Sig. (2-tailed)		0.661	0.036	0.881	0.653	0.378	0.254
No. of samples	N	10	10	10	10	10	10	10

*Correlation is significant at the 0.05 level (2-tailed)

With reference to Table 15a, the Pearson’s product movement correlation coefficient of the tested variable 0–30 cm were calculated between elevation and the heavy metals. Copper, Iron, Zinc, Lead, Arsenic and Cadmium had a correlation coefficient of − 0.085, 0.173, 0.145, 0.525, 0.04 and 0.658, respectively. Lead (Pb) and Cadmium (Cd) showed high positive relationship with the elevation. Lead is a very important component in battery production, from the result of this study it is shown that lead is a very important material generated on daily activities being carried out on the study area. Cadmium is important metal which are found in batteries, pigments, metal coating, plastics, and alloys, this is also one of the important waste components generated within the study area. The elevation-metal relationship shows that these materials are found more in areas of high elevation being mostly areas of worksites. Copper (Cu) in the other hand shows weak negative relationship indicating material settlements on areas of low and flat elevation. Copper are essential components in automobile repairs and as found useful in components like wiring, radiators, connectors, brakes and bearing. Iron (Fe) shows weak positive relationship with elevation, this shows that elevation barely contributes to the distribution of iron found in the study area soil^100^.

With reference to Table 15b, the Pearson’s product movement correlation coefficient of the tested variable 30–60 cm was calculated between elevation and the heavy metals with Copper, Iron, Zinc, Lead, Arsenic and Cadmium scoring 0.159, 0.663, 0.055, − 0.163, 0.313 and − 0.398 respectively. Iron (Fe) shows strong positive relationship with elevation and this shows that iron is not easily infiltrated into the soil and therefore are found more on the surface soils of the study area surface. Lead (Pb) and Cadmium (Cd) shows negative relationship with the elevation. Lead and Cadmium are very important component in battery production, from the result of this study it is shown that lead is a very important waste material generated on daily activities being carried out on the study area. Lead and Cadmium shows more infiltration capability and therefore are found more at soil level between 30 and 60 cm. The elevation-metal relationship shows that Cd and Pb are found more in areas of low elevation in the study area. Copper (Cu) on the other hand shows weak positive relationship and this mean it is found mostly on the surface soil of the study area. Copper are essential components in automobile repairs and is found useful in components like wiring, radiators, connectors, brakes and bearing. Zinc has no relationship with elevation according to the result test^101^.

## Conclusions and recommendations

### Conclusions

The presence heavy metals of Copper (Cu), Iron (Fe), Zinc (Zn), lead (Pb), Arsenic (As) and Cadmium (Cd) were detected in low concentration within and around the Nsukka auto-mechanics workshop cluster. At the depth of 0–30 cm the mean concentrations of Cu, Fe, Zn, Pb, As and Cd were 0.751 ± 0.152, 3.327 ± 0.886, 23.464 ± 3.818, 1.115 ± 0.138, 2.912 ± 0.734 and 0.365 ± 0.323 mg/kg, respectively while, outside the cluster (control) at the same depth, the concentrations of Cu, Fe, Zn, Pb, As and Cd were 0.6875 ± 0.114, 2.516 ± 0.577, 12.841 ± 1.847, 0.903 ± 0.229, 1.911 ± − 0.494 and 0.097 ± 0.023 mg/kg respectively. From the depth of 30–60 cm the concentrations of Cu, Fe, Zn, Pb, As and Cd were 0.716 ± 0.069, 2.981 ± 0.664, 30.113 ± 3.013, 1.21 ± 0.158, 2.6 ± 0.515 and 0.133 ± 0.018 mg/kg respectively. At the same depth outside the cluster, the respective values were 0.611 ± 0.115, 2.233 ± 0.417, 15.736 ± 1.546, 0.887 ± 0.219, 1.669 ± 0.362 and 0.091 ± 0.042 mg/kg. All the studied heavy metals fell within the limit when compared to WHO standard.

Statistically, to evaluate the difference in heavy metal concentrations between samples from inside and outside the clusters, we conducted an analysis of variance (ANOVA) test. The results showed a significant gap in heavy metal levels between samples collected within and outside the auto mechanic cluster, with a p-value < 0.05. This finding suggests that activities associated with auto mechanics within the cluster significantly affect heavy metal levels compared to areas beyond the cluster.

Moreover, Soil pollution assessment involved the application of indices such as the Geo-accumulation Index (Igeo), Contamination factor (Cf), and anthropogenic metal concentration (QoC). Zinc, Cadmium, and Arsenic exhibited the most elevated contamination factors, signifying substantial soil pollution likely attributed to human activities.

Generally, from the spatial interpolation technique used, kriging method performed better than spline because spline has more significant different values compared to kriging. This was also proved by Levene’s Independent Test. There was a relatively high correlation between the concentration of Pb (R^2^ = 52%) and Cd (R^2^ = 65%) and elevation of the sampling points at 0–30 cm depths. At 30–60 cm depth, only Fe showed some correlation (R^2^ = 66.3%). This study has established the concentration levels of heavy metals in Nsukka auto-mechanic village which provides background information for further studies in the study area. Spatial map of heavy metal levels of the auto mechanic workshop cluster was also developed.

### Recommendations

Having established heavy metals concentration in the study area, this study has provided background information for monitoring of increased levels of heavy metal as well as for further studies. The low levels of the some of the metals when compared to some other studies should be re-evaluated for the possible presence of bio-remediating materials in the study area. Further studies should be carried out on the crops and vegetation of the study area and should be used to determine their heavy metal uptake, which may be another remediating media. Possible effect on groundwater was not within the scope of this therefore, it is recommended for further studies.

## Data Availability

All data generated or analyzed during this study are included in this published article.
